# The Effects of Methylphenidate on Decision Making in Attention-Deficit/Hyperactivity Disorder

**DOI:** 10.1016/j.biopsych.2008.04.017

**Published:** 2008-10-01

**Authors:** Elise E. DeVito, Andrew D. Blackwell, Lindsey Kent, Karen D. Ersche, Luke Clark, Claire H. Salmond, Anna Maria Dezsery, Barbara J. Sahakian

**Affiliations:** a Department of Psychiatry, University of Cambridge, Cambridge, United Kingdom; b Section of Child and Adolescent Psychiatry, University of Cambridge, Cambridge, United Kingdom; c Behavioral and Clinical Neuroscience Institute, University of Cambridge, Cambridge, United Kingdom

**Keywords:** Attention-deficit/hyperactivity disorder (ADHD), Cambridge Gamble Task (CGT), decision making, methylphenidate (MPH)

## Abstract

**Background:**

Children with attention-deficit/hyperactivity disorder (ADHD) frequently display poor judgment and risk taking in their everyday behavior, but there are little empirical data on decision-making cognition in this disorder. The objectives of the study were to assess the effects of stimulant medication on decision making in ADHD and compare performance on the Cambridge Gamble Task between boys with and without ADHD.

**Methods:**

Twenty-one boys (aged 7–13) diagnosed with ADHD underwent a double-blind, placebo-controlled trial of methylphenidate (.5 mg/kg) during which they performed the Cambridge Gamble Task (CGT). A healthy age-matched control group was tested on two occasions off drug.

**Results:**

The ADHD group bet more conservatively on the methylphenidate session than on the placebo session. In comparison with healthy control subjects, the ADHD group made more poor decisions, placed their bets more impulsively, and adjusted their bets less according to the chances of winning. Poor decision making was correlated with parent-reported symptoms and disruptive behavior in the ADHD group.

**Conclusions:**

Methylphenidate reduced risk-prone betting behavior on the CGT. Compared with control subjects, children with ADHD display a number of decision-making deficits on the task, and the measure of rational decision making may serve as an ecologically valid neuropsychological marker of impairment.

Attention-deficit/hyperactivity disorder (ADHD), a prevalent psychiatric disorder characterized by hyperactivity, impulsivity, and inattention, is diagnosed by pervasive maladaptive behaviors during childhood (1). Children with ADHD display a range of cognitive impairments on laboratory tasks and risk-taking behaviors in daily life. Previous neuropsychological studies have investigated the mechanisms underlying ADHD behavior in terms of disinhibition, delay aversion, and abnormal reward sensitivity (2). However, few studies have assessed affective decision making. Iowa Gambling Task (IGT) findings are inconsistent in ADHD, possibly reflecting task sensitivity to disrupted working memory and learning (3,4).

Pharmacotherapy with methylphenidate (MPH) improves behavioral symptoms and cognitive function (e.g., attention, inhibition, working memory) in ADHD (5), producing similar effects in animals and healthy humans (6). Methylphenidate inhibits dopamine and noradrenaline reuptake, primarily in the prefrontal cortex (PFC) (6) and may compensate for frontostriatal pathophysiology in ADHD. The effect of MPH treatment on decision-making cognition in ADHD is not known, although impulsive and risk-taking behaviors are important aspects of ADHD symptomatology. We investigated methylphenidate's impact on decision making in childhood ADHD, using the Cambridge Gamble Task (CGT). Cambridge Gamble Task measures decision making and risk taking through betting behavior. The CGT was devised to minimize working memory and learning components by presenting outcome probabilities explicitly (7). Neural circuitry implicated in emotional decision making continues to develop through adolescence (8), so children may perform CGT differently than adults. As this is the first study to employ the CGT in this age group, we included a group of healthy boys matched for demographic variables to detect decision-making cognition abnormalities in ADHD. The relationship between CGT performance and behavioral ratings was assessed to determine the task's ecological validity.

## Methods and Materials

Parental written informed consent and ethics committee approval were obtained.

Psychiatrists referred consecutive attendees to a childhood ADHD outpatient clinic. Diagnoses following DSM-IV guidelines including pervasiveness of symptoms (1) were established with 3-hour clinical assessments based on the Schedule for Affective Disorders and Schizophrenia for School-Age Children (K-SADS), developmental and family histories, and teacher reports. Attention-deficit/hyperactivity disorder patients (*n* = 21) were males, aged 7 to 13, and stabilized on methylphenidate with no primary learning disabilities or concomitant neurological, psychiatric, or behavioral disorders (except history of oppositional defiant disorder; *n* = 14). A healthy control group (HC) (*n* = 22; aged 7 to 12) was recruited with posters from the local community.

The ADHD boys underwent a double-blind, placebo-controlled, crossover design of a single .5 mg/kg dose of methylphenidate or placebo. One child received .25 mg/kg (10 mg) due to his high weight and low therapeutic dose. Participants abstained from MPH for 21 to 28 hours (approximately 5 to 7 half-lives) prior to testing sessions. Methylphenidate reaches peak plasma concentration in approximately 2 hours (9). Questionnaires were completed once at the start of a visit to avoid treatment effects. Cambridge Gamble Task testing began at least 1.75 hours after pills were ingested. Healthy control subjects attended two sessions but received no pills.

The Mood and Feeling Questionnaire (10) measured depressive symptoms. Parents completed disruptive behavior questionnaires (Achenbach Child Behavior Checklist [11]; Conners Symptom Behavior Checklist [12]) based on their son's behavior without medication. The ADHD group completed Visual Analogue Scales (VAS) (13) modified with age-appropriate vocabulary prior to pills (t_0_), prior to cognitive testing (t_1_), and after testing (t_2_).

The Cambridge Gamble Task (Cambridge Neuropsychological Test Automated Battery [CANTAB]; www.camcog.com) (7) assessed decision making under risk. On each trial, participants were presented with an array of 10 boxes, colored red or blue. The ratio of colored boxes varied across trials. On each trial, the participant was asked to guess which color concealed a token, then wager a proportion of his total points on his color decision. Wagers were offered in ascending (5%, 25%, 50%, 75%, 95% of current points) or descending (95%, 75%, 50%, 25%, 5% of current points) sequences presented for 2.5 seconds each. After the bet was placed, the hidden token was revealed and the bet was added to or subtracted from the running score. “Rational choices” is the proportion of trials where the majority color was chosen. “Deliberation time” is the latency to make the color choice. “Amount bet” is averaged across conditions and box ratios. Higher bets are assumed to indicate risk preference. “Impulsivity index” is the difference in percentage bet in descending versus ascending conditions. Consistently early bets (e.g., 95% points descending − 5% points ascending) produce a high impulsivity index. “Risk adjustment index” quantifies bet calibration across ratios {[2*(% bet 9:1) + (% bet 8:2) − (% bet 7:3) − 2*(% bet 6:4)]/Average % bet}, so higher scores are preferable (14).

Repeated-measures analysis of variance (ANOVA), with between-subject factors of methylphenidate/placebo order, compared the ADHD group on placebo (ADHD-P) versus methylphenidate (ADHD-MPH) on CGT measures and change in VAS factors (t_1_+ t_2_− t_0_) (13). Deliberation times were logarithmically transformed; rational choices were arcsine transformed (14) to decrease skew and stabilize variances. Groups were compared on demographic variables with *t* tests and chi-squared analysis. Univariate analysis of covariance (ANCOVA), covaried for age, examined ADHD-P versus HC performance on all CGT measures and ADHD-MPH versus HC for measures that significantly responded to drug manipulation. Healthy control group data were assessed for practice effects using repeated measures ANOVA then averaged across visits, since ADHD-P visits were counterbalanced. Effect sizes were calculated as *d* = (μ_1_− μ_2_)/√[(σ_1_^2^ + σ_2_^2^)/2] (15). The relationship between CGT measures and behavioral ratings (Conners_Total_, Achenbach_Total_, Achenbach_Internalizing_, Achenbach_Externalizing_) were determined using Pearson's product-moment correlation coefficient. Other summary questionnaire measures were not analyzed.

## Results

Attention-deficit/hyperactivity disorder and HC groups were matched for age (*t* = −.57, *p* = .571; mean_ADHD_ = 10.00, standard deviation_ADHD_ = 2.05; mean_HC_ = 10.32, standard deviation_HC_ = 1.59), test order (*t* = −.45, *p* = .659), days between visits (*t* =− .71, *p* = .482), years of education (*t* = −1.06, *p* = .297), and distribution of younger (7–10) versus older (11–13) children (*df* = 1, *X*^2^ = 1.13, *p* = .29). The ADHD group had higher disruptive behavior ratings (Achenbach_Total_ *t* = 6.80, *p* < .001; Achenbach_Internalizing_ *t* = 4.73, *p* < .001; Achenbach_Externalizing_ *t* = 6.79, *p* < .001; Conners_Total_ *t* = 9.06, *p* < .001) and a trend toward higher depressive symptoms (*t* = 1.88; *p* = .068). The HC group showed no significant practice effects on the CGT (*F* = 1.43–2.63, *p* = .13–.25, *d* = .19–.49).

**Figure 1:**
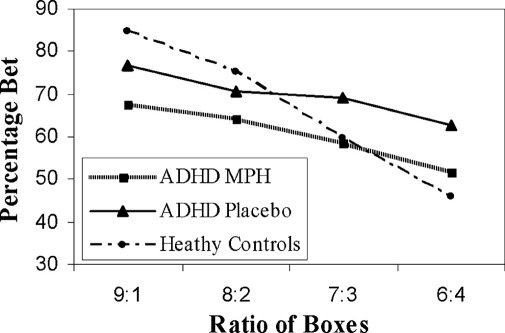
Methylphenidate reduces the amount bet by ADHD group without ameliorating risk adjustment deficits relative to control subjects. Bets are displayed as an average of the percentage of total points wagered on each decision. ADHD, attention-deficit/hyperactivity disorder; MPH, methylphenidate.

On methylphenidate, the ADHD group bet significantly fewer points than on placebo (Figure 1) but did not differ on other CGT measures (Table 1). Methylphenidate did not have a significant main effect on VAS factors (calmness: *F* = 1.20, *p* = .291; alertness: *F* = .60, *p* = .450; happiness: *F* = .33, *p* = .576). Participants receiving methylphenidate at visit 1 reported feeling calmer than on placebo (*F*_drug × visit_ = 8.26, *p* = .012).

The ADHD-P group performed less optimally than HC on rational choices across all box ratios, risk adjustment, and impulsivity index but did not differ on amount bet or deliberation times (Figure 1, Table 1). The ADHD-MPH and HC groups did not significantly differ on amount bet (*F* = 2.96, *p* = .09, *d* = .53).

**Figure 2:**
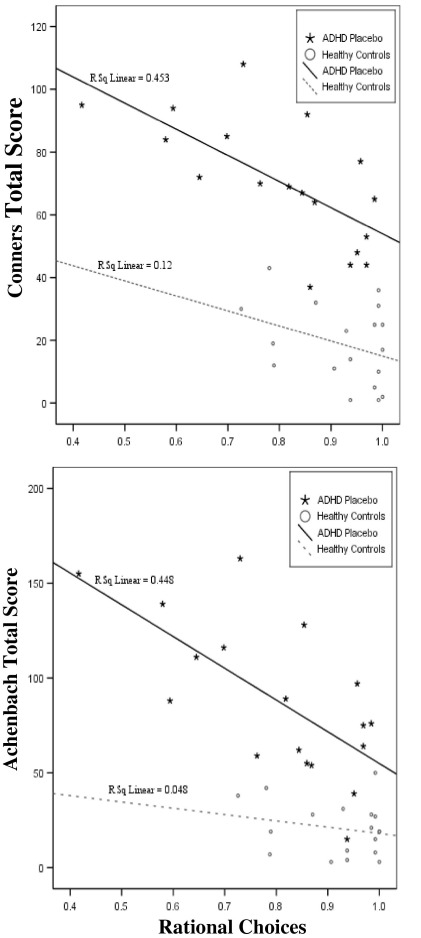
Rational choices and behavioral ratings. Rational choices is the average for the proportion of trials where the subject selected the box color that was in the majority. ADHD, attention-deficit/hyperactivity disorder.

For ADHD-P, higher behavioral ratings were associated with poor quality of decision making (Conners_Total_ *r*_18_ =−.673, *p* = .002; Achenbach_Total_ *r*_18_ =−.670, *p* = .002; Achenbach_Internalizing_ *r*_18_ =−.506, *p* = .038; Achenbach_Externalizing_ *r*_18_ =−.545, *p* = .024) (Figure 2). For HC, Achenbach correlated with impulsivity index (Achenbach_Total_ *r*_19_ = .545, *p* = .016; Achenbach_Externalizing_ *r*_19_ = .701, *p* = .001). No other behavioral rating scores correlated significantly with CGT performance (*p* ≥ .195), although low variance on rating scales in control subjects may have limited our sensitivity to detect additional correlations.

## Discussion

Methylphenidate significantly reduced CGT betting behavior in children with ADHD without affecting performance on other measures. These methylphenidate effects replicated findings in frontal variant frontotemporal dementia (fvFTD) patients, where MPH reduced betting (16). The ventromedial prefrontal cortex (vmPFC) has been robustly linked to decision making: patients with vmPFC disruption (e.g., fvFTD) display elevated betting on CGT (3,16). It is conceivable that MPH modulates betting behavior via action on the neural network, including vmPFC, which subserves performance on CGT.

High CGT betting increases potential losses, therefore indicating risk taking. Despite their abnormal pattern of betting, the ADHD group did not bet high amounts for all ratios. However, case-control comparisons indicated significant decision-making impairments in the ADHD group on other CGT variables. On placebo, the ADHD group bet more impulsively than control subjects, selecting high or low bets early in the bet sequence. This shortens the task duration, consistent with ADHD performance profiles on delayed reward tasks, indicating delay aversion (17) or motor impulsivity. The ADHD-P group also made fewer rational choices and risk adjusted less than healthy control subjects, yet both groups altered their bets according to the ratios and made rational choices at above-chance rates. The magnitude of these deficits on rational choices was associated with higher ADHD symptom ratings. In this ADHD sample, CGT rational choices accounted for even more of the variance in behavioral symptoms than was accounted for by delayed choice or stop signal impulsivity in a previous study (17). Replication and extension of these findings in a larger ADHD sample size including female subjects would clarify their generalizability. This measure of poor decision making was previously found to correlate with key clinical measures in other disorders associated with PFC abnormalities (e.g., 7).

Several factors may underpin the deficits in rational choices and risk adjustment. Children with ADHD modulate their behavior according to rates and magnitudes of gains and losses less than their peers (4), who do so less than adults (18). The ability to weigh competing factors to optimize decision making develops into adulthood (19). Therefore, poor risk adjustment in ADHD could represent an exaggeration of a normal developmental pattern (20).

## Conclusions

Poor quality decision making was associated with ADHD behavioral symptoms. Methylphenidate induced conservative betting behavior in childhood ADHD but did not ameliorate their decision-making deficits.

## Figures and Tables

**Table 1:** Cambridge Gamble Task Key Measures

CGT Measures	HC^a^^b^	ADHD-P^a^	ADHD-MPH^a^	Group Effect^c^	Drug Effect^d^
Rational Choices	.93 ± .02, (.90, .94)	.80 ± .04	.81 ± .04	10.65 (.002)^e^, .98	.00 (.989), .03
Deliberation Time	2517 ± 390, (2611, 2424)	3203 ± 348	2778 ± 253	1.25 (.273), .44	.96 (.342), .28
Amount Bet	66.67 ± 2.10, (65.85, 69.68)	69.80 ± 3.13	60.50 ± 2.91	.66 (.422), .27	8.54 (.010)^e^, .68
Impulsivity Index	18.34 ± 3.05, (26.10, 18.35)	31.44 ± 5.56	33.25 ± 5.76	4.44 (.042)^e^, .67	.14 (.717), .13
Risk Adjustment	1.48 ± .19, (1.37, 1.59)	.44 ± .31	.54 ± .21	11.38 (.002)^e^, .93	.37 (.551), .10

ADHD, attention-deficit/hyperactivity disorder; ADHA-MPH, ADHD group on MPH; ADHD-P, ADHD group on placebo; CGT, Cambridge Gamble Task; HC, healthy control; MPH, methylphenidate; SEM, standard error of the mean.

a Means ± standard error of the mean (SEM) are presented for Cambridge Gamble Task (CGT) measures; proportion of rational choices, deliberation time (msec), amount bet, impulsivity index, and risk adjustment.

b Healthy control (HC) data are averaged across both visits. Means for each visit are also provided (mean_HCvisit1_, mean_HCvisit2_).

c Group effect notes the differences and effect sizes [*F* (*p*), *d*] between the healthy control data averaged across both visits and the ADHD group on placebo, covaried for age.

d Drug effect describes significant differences and effect sizes [*F* (*p*), *d*] between the drug (ADHD-MPH) and placebo (ADHD-P) conditions in the ADHD group with a between-subject factor of drug/placebo visit order.

e Statistically significant *p* values.
